# Influence of Doping and Nanostructuration on n-Type Bi_2_(Te_0.8_Se_0.2_)_3_ Alloys Synthesized by Arc Melting

**DOI:** 10.1186/s11671-016-1823-9

**Published:** 2017-01-17

**Authors:** Mouna Gharsallah, Federico Serrano-Sanchez, Norbert M. Nemes, Jose Luis Martinez, Jose Antonio Alonso

**Affiliations:** 1 Instituto de Ciencia de Materiales de Madrid, C.S.I.C., Cantoblanco, E-28049 Madrid, Spain; 2 Sfax University, National School of Engineers, Sfax, BPW 3038 Tunisia; 3 Departamento de Física de Materiales, Universidad Complutense de Madrid, E-28040 Madrid, Spain

**Keywords:** Thermoelectrics, Nanostructuration, Lattice thermal conductivity, Bismuth telluride, Neutron powder diffraction

## Abstract

In competitive thermoelectric devices for energy conversion and generation, high-efficiency materials of both n-type and p-type are required. For this, Bi_2_Te_3_-based alloys have the best thermoelectric properties in room temperature applications. Partial replacement of tellurium by selenium is expected to introduce new donor states in the band gap, which would alter electrical conductivity and thermopower. We report on the preparation of n-type Bi_2_(Te_1-x_Se_x_)_3_ solid solutions by a straightforward arc-melting technique, yielding nanostructured polycrystalline pellets. X-ray and neutron powder diffraction was used to assess Se inclusion, also indicating that the interactions between quintuple layers constituting this material are weakened upon Se doping, while the covalency of intralayer bonds is augmented. Moreover, scanning electron microscopy shows large surfaces perpendicular to the c crystallographic axis assembled as stacked sheets. Grain boundaries related to this 2D nanostructuration affect the thermal conductivity reducing it below 0.8 Wm^−1^K^−1^ at room temperature. Furthermore, Se doping increases the absolute Seebeck coefficient up to −140 μV K^−1^ at 400 K, which is also beneficial for improved thermoelectric efficiency.

## Background

In a sustainable-energy environment, the conversion between thermal and electrical energy carried out by thermoelectric materials has become essential. Thermoelectric devices present several advantages such as reliability, absence of mobile parts and durability. Such devices shall find a large spectrum of applications ranging from refrigeration to waste heat recovery, temperature measurements and thermal energy detection [1–6]. The performance of thermoelectric materials is evaluated by the figure of merit (ZT) defined as:

$$ \mathrm{Z}\mathrm{T} = T\ {S}^2\sigma /\kappa $$

where *T* is the average absolute temperature, *S* is the Seebeck coefficient, *σ* is the electrical conductivity, and *κ* is the total thermal conductivity.

Thus, on the path towards more efficient thermoelectric materials, it is necessary to achieve the best compromise between these three intrinsic physical quantities *S*, *σ*, and *κ*. In thermoelectric research, this challenging task is broached through many different ways. Band engineering, hierarchical architectures, complex crystal structures, and rattling semiconductors are amongst recent approaches for ZT improvement [7–10]. Nanostructuration also plays an essential role, as there are many theoretical and experimental works showing enhanced thermoelectric efficiency in bulk nanostructured and nanosized materials, where the relevant physical properties may be decoupled [11–13].

Most thermoelectric materials are heavily doped narrow band-gap semiconductors where the concentration of charge carriers has been optimized to offer a high electric conductivity while maintaining elevated Seebeck coefficients and low electronic thermal conductivity. Bismuth chalcogenides and, in particular, Bi_2_Te_3_ and its solid solutions, are known as the most efficient thermoelectric materials for near room temperature applications [5, 14]. Their thermoelectric performance is notably affected by their exact stoichiometry, as Te and Bi vacancies and lattice (antisite) defects directly vary the carrier concentration.

Fine adjustment of the carrier concentration is done mainly by doping and controlling the synthesis conditions. Diverse methods are employed for the preparation of thermoelectric materials, from physical methods, such as high-energy ball milling, melting, and hot pressing, to wet chemical methods, such as polyol synthesis of nanoparticles [15]. Present in all these methods, after the prior preparation of the material, is a compaction step and/or steps of conditioning, comprising time-consuming processes, and expensive instrumentation.

Thermoelectric properties of bismuth telluride-related compounds are usually optimized by various technical processes, such as ionic and element substitution [16–18], changes in the intricate macro- and micro-structure [19–26] and diverse variations in the synthesis conditions [27–29]. The inclusion of Se in bismuth telluride-type compounds, constituting Bi_2_Te_3_-Bi_2_Se_3_ solid solutions, enlarges the band-gap energy by stronger Se-Bi interactions and creates new donor levels close to the bulk band gap, which may increase the electrical conductivity [30–32], thus enhancing the thermoelectric performance. Furthermore, doping-induced point defects, such as atomic mass fluctuation and lattice deformation, yield a reduction in the lattice thermal conductivity. Besides, nanostructured composites present low-energy electron filtering and enhanced phonon scattering [11, 33] at the interfaces, although a strong drawback has been found in Se-doped polycrystalline samples as this improvement is countered by deteriorating electrical conductivity. This effect is attributed to the higher sensitivity of n-type Bi_2_Te_3 − *x*_Se_*x*_ to the lattice directions than the parent compound and p-type Bi_2 − *x*_Sb_*x*_Te_3_, for which bulk samples commonly present randomly orientated grains; therefore, many efforts are still to be made to achieve bulk nanostructured samples with increased phonon scattering while keeping a preferential orientation to maintain the power factor (defined as the product of *S* ^2^ and *σ*) [34, 35].

Recently, we have reported on a direct method to synthesize highly nanostructured Bi_2_Te_3_ samples in short reaction times, in the form of robust pellets directly usable in devices [36]. The structural characterization showed a near-perfect stoichiometry and an important anisotropy of the atomic displacement factors. Electrical conductivity was notably improved while thermal conductivity was not enlarged. Based on these results, we prepared doped samples for the optimization of the thermoelectric parameters.

In this paper, we describe the preparation of n-type Bi_2_Te_2.4_Se_0.6_ by the same straightforward arc-melting procedure. We found a huge preferred orientation and nanostructuration while a decrease in thermal and electrical conductivity is observed. The sample was structurally characterized by X-ray diffraction (XRD) and neutron powder diffraction (NPD), since neutrons provide a bulk analysis and avoid preferred orientation problems. A microscopic study of the nature of the raw material was realized by SEM, and the three thermoelectric properties (Seebeck, electrical, and thermal conductivity) were measured.

## Methods

The n-type Bi_2_[Te_0_._8_Se_0_._2_]_3_ alloy was synthesized in an Edmund Buhler mini-arc furnace using direct arc melting in a water cooled copper crucible with a tungsten electrode under purified argon atmosphere. The starting materials were pure elements of Bi (99.999%, Cerac), Te (99.999%, Alfa Aesar), and Se (99.95%, Alfa Aesar) that were weighted and mixed according to the stoichiometric ratio. The mixed powders were put into pellets and molten and re-molten four times to promote homogenization. It was also necessary to work in a slight argon overpressure and to carry out several argon rinsing cycles in order to purify the atmosphere bell. In order to minimize the evaporation effects, we reduced the melting time and controlled the arc power supply to use the lowest current that melts the mixture. After melting, the ingot was ground to powder several times in an agate mortar previous to structural characterization.

Structural phase analysis was carried out using X-ray diffraction (XRD) by Cu Kα radiation on a Bruker-AXS D8 diffractometer (40 kV, 30 mA), run by DIFFACT^PLUS^ software, in Bragg-Brentano reflection geometry with Cu Kα radiation (*λ* = 1.5418 Å). The data were collected by 0.04 steps over a 2*θ* range from 10° to 64°.

The structure, phase purity, and homogeneity of the elaborated sample were checked by NPD. The crystallographic structure was refined from a high-resolution NPD pattern collected at the HRPT diffractometer of the SINQ neutron source at the Paul Scherer Institute in Villigen (Switzerland), with a wavelength *λ* = 1.494 Å. Cylindrical vanadium holders were used to pack the samples (diameter 8 mm), counting during 2 h in the high-intensity mode; the sample holder was rotating during the acquisition time. The diffraction data were analyzed by the Rietveld method using the FULLPROF program [37]. The line shape of the diffraction peaks was generated by a Thompson-Cox-Hastings pseudo-Voigt function. 8.532, 5.800, and 7.970 fm were, respectively, the coherent scattering lengths used for Bi, Te, and Se. A preferred orientation correction accounting for platelets perpendicular to [001] direction was added to the refinement. There were no regions excluded in the refinement. In the final runs, the following parameters were refined: scale factor; background coefficients; zero-point error; pseudo-Voigt corrected for asymmetry parameters; occupancy of Bi, Te, and Se; positional coordinates; and anisotropic displacements for all the atoms. Superficial analysis by FE-SEM was performed in a FEI-Nova microscope.

Measurements of thermoelectric transport properties were carried out in a commercial system (physical property measurement system (PPMS) by Quantum Design). The measurements were made in the residual vacuum of He atmosphere, under a pressure of 10^−5^ Torr, in the temperature range of 2 to 400 K. Disks of 10 mm diameter, featuring perfectly parallel faces, were obtained by treatment of the as-grown ingots under an isostatic pressure of 10^3^ psi. These disks where then cut with a diamond saw to bar-shaped specimens. The size of the n-Bi_2_Te_2.4_Se_0_._6_ pellets were typically 10 × 3 × 1.5 mm^3^ with four Cu wires attached with EPO-TEK® H20E paste. Thermoelectric properties were measured perpendicular to the pellet pressing direction. Throughout the whole temperature range, a temperature gradient of 3% was used.

The Hall coefficient was measured using the resistivity option of the PPMS system in an approximate van der Pauw geometry, in the disk-shaped pellets with spring-loaded pins for contacts. The charge carrier concentration was determined via the relation *n* = −1/*R*_H_e^−^ from the Hall coefficient *R*_H_.

## Results and discussion

### Crystal structure

**Fig. 1:**
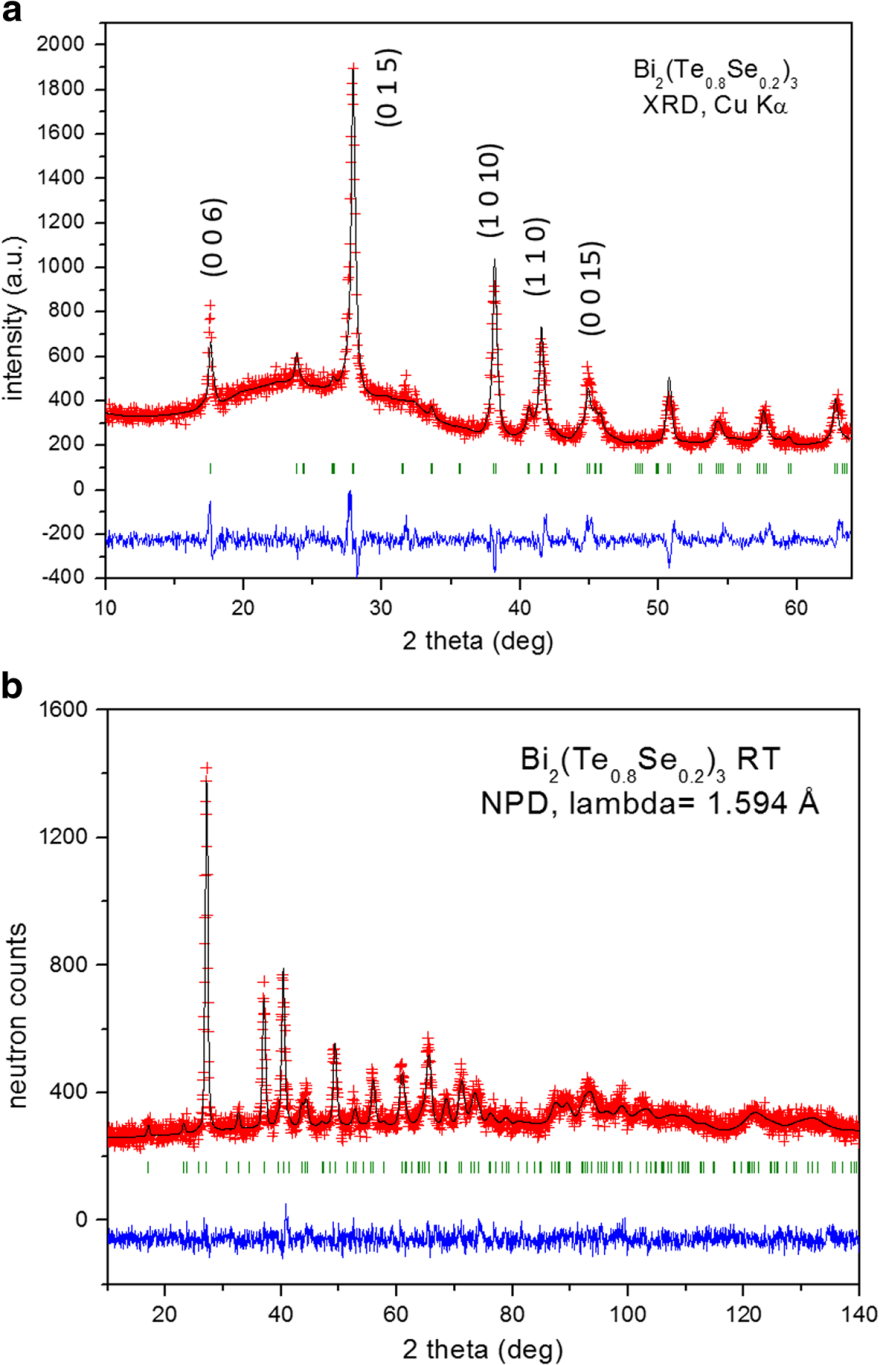
**a** Pattern from XRD data for ground n-Bi_2_Te_2.4_Se_0_._6_, showing refinement by the Rietveld method in the space group R-3m. There is a notable preferred orientation, increasing the [001] reflections. **b** Calculated (*full line*), difference (*at the bottom*), and observed (crosses) neutron powder diffraction patterns for n-Bi_2_Te_2.4_Se_0.6_ at 298 K

A representative Rietveld refinement of the XRD pattern for n-type Bi_2_Te_2.4_Se_0.6_ (Fig. 1a) shows a Bi_2_Te_3_-type structure, defined in the space group R-3m. Patterns show a strong preferred orientation enhancing the (0 0 *l*) reflections, pointing to a strongly textured as-grown sample. In order to improve the match between observed and simulated profiles, a preferred orientation function was introduced as correction during the profile refinement.

Crystallographic analysis by neutron powder diffraction (NPD) allows investigating the essential structural details of Bi_2_Te_2.4_Se_0.6_, including the anisotropic displacement factors. Preferred orientation effects are largely eliminated by the measurement conditions: neutrons can penetrate bulk amounts of material, which are ground to powder, packed into vanadium cylinders that are furthermore rotating continuously during data collection. Also, the lack of form factor in neutron diffraction means that high-angle diffraction peaks are also well resolved, which allows determining precisely the anisotropic displacement factors. Refinement of NPD data at RT of the crystal structure was carried out in the Bi_2_Te_3_-type model [38] defined in the hexagonal setting of the rhombohedral R-3m space group (no. 12), *Z* = 3, with Bi located at 6*c* (0 0 *z*) Wyckoff site and the two types of tellurium and selenium, Te1/Se1 at 3*a* (0 0 0) positions and Te2/Se2 at 6*c*. Calculated profiles present an excellent agreement with the experimental data (Fig. 1b). A minor preferred orientation correction effectively improved the refinement for all the reflections. Table 1 presents the main atomic parameters, displacements factors, and discrepancy factors resulting from the refinement. Unit-cell parameters are *a* = 4.3315 (4) and *c* = 30.208 (5) Å. Unit-cell size is significantly reduced from that of the parent Bi_2_Te_3_ compound (with unit-cell parameters: *a* = 4.385915 (6), c = 30.495497 (1) Å, [35]), which can be understood as a consequence of the smaller ionic size of Se^2−^ vs Te^2−^.

**Table 1:** Structural parameters for Bi_2_(Te_0.8_Se_0.2_)_3_ refined in the R-3m space group (hexagonal setting) from NPD data collected at RT. ​The discrepancy factors after the refinement are also included

Fractional atomic coordinates and equivalent isotropic displacement parameters						
	Wickoff site	*x*	*y*	*z*	*U*_eq_	Occ. (<1)
Bi	6*c*	0.00000	0.00000	0.3956 (4)	0.009 (4)	
Te1	3*a*	0.00000	0.00000	0.00000	0.025 (11)	0.80 (9)
Se1	3*a*	0.00000	0.00000	0.00000	0.025 (11)	0.20 (9)
Te2	6*c*	0.00000	0.00000	0.7897 (3)	0.017 (8)	0.79 (3)
Se2	6*c*	0.00000	0.00000	0.7897 (3)	0.017 (8)	0.21 (3)
Anisotropic displacement parameters (Å^2^)						
	*U* ^11^	*U* ^22^	*U* ^33^	*U* ^12^	*U* ^13^	*U* ^23^
Bi	0.009 (2)	0.009 (2)	0.009 (8)	−0.005 (2)	0.00000	0.00000
Te1	0.020 (7)	0.020 (7)	0.04 (2)	−0.010 (7)	0.00000	0.00000
Se1	0.020 (7)	0.020 (7)	0.04 (2)	−0.010 (7)	0.00000	0.00000
Te2	0.016 (5)	0.016 (5)	0.019 (12)	−0.008 (5)	0.00000	0.00000
Se2	0.016 (5)	0.016 (5)	0.019 (12)	−0.008 (5)	0.00000	0.00000
Discrepancy factors						
*R*_p_ = 2.69%, *R*_wp_ = 5.89%, *R*_exp_ = 5.36%, *χ* ^2^ = 1.21, *R*_Bragg_ = 5.15%						

Unit-cell parameters: *a* = 4.3315 (4) Å and *c* = 30.208 (5) Å, *V* = 490.9 (1) Å^3^, *Z* = 3

**Fig. 2:**
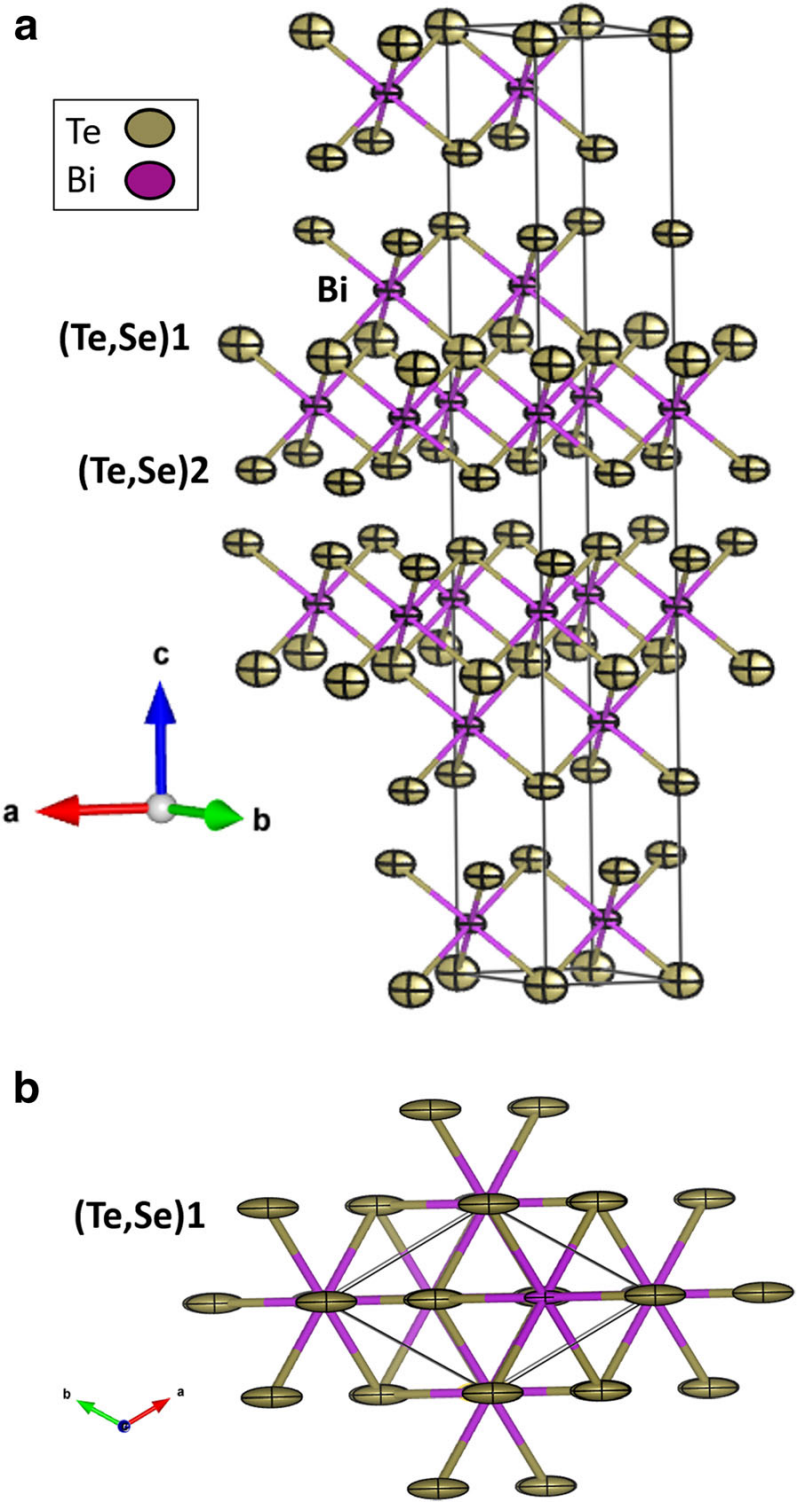
**a** View of the crystal structure of n-Bi_2_Te_2.4_Se_0_._6_ showing anisotropic atomic displacement factors as ellipsoids directed along [1 1 0] direction, within the plane of covalent layers. **b** View along [0 0 1] direction

Figure 2 shows two views of the crystal structure of Bi_2_Te_2.4_Se_0.6_. Similar to Bi_2_Te_3_, it is made up of hexagonal close-packed sheets of a series of quintuple layers with a stacking sequence of covalently bonded A2-Bi-A1-Bi-A2 atoms (*A* = Te or Se), as shown in Fig. 2a. The interlayer forces between quintuple layers (A2-A2 interactions) are principally weak van der Waals type. Therefore, crystals of these compounds are easily cleaved parallel to ab plane. Interestingly, Se atoms were found, by the NPD Rietveld refinement, to be randomly distributed at both Te sublattices. This is somewhat contrary to expectations, as there is a slight difference in the chemical environment of Te1 and Te2 which gives Bi-Te1 bond a minor ionic component, favoring that Se would first preferentially replace Te2 sites [39], immediately followed by random introduction of Se at Te1 sites. Terminal Te2/Se2 atoms are covalently bonded to three Bi atoms at 3.050 (9) Å, with the non-bonding electron pairs directed to the interlayer spacing, while Te1/Se1 is coordinated to six Bi atoms in octahedral sites at distances of 3.161 (7) Å. Bi is coordinated to 3 Te1/Se1 plus 3 Te2/Se2 forming a distorted octahedron. Additionally, the analysis of the neutron data yielded accurate anisotropic displacement factors for all the atoms. Figure 2b, in particular, shows the elongated ellipsoids of (Te, Se)1 directed along the cell diagonal [1 1 0] direction. The refinement of the occupancy factors of Bi, Se, and Te stoichiometry is obtained with standard deviations (±0.02). One of the strengths of this study, using NPD, is that we can give a bulk characterization of any possible off-stoichiometry. The stoichiometry change due to evaporation loss of the elements is thus evaluated by NPD, refining the occupation factor of each atom position. Despite the possible evaporation of the various elements during arc melting, NPD shows practically the same ratio as the weighed elements before the synthesis, in our process.

It is interesting to compare these results with similar structural refinements recently published for pristine Bi_2_Te_3_ [36]. We observe that both Bi-Te1 distances (3.253 Å) and Bi-Te2 distances (3.061 Å) decrease upon Se introduction, which is an additional indication that Se doping occurs at both un-equivalent positions. Regarding the connections established between the quintets, of van der Waals type (Te_2_/Se_2_-Te_2_/Se_2_), the distance is 3.691 (9) Å vs 3.660 (6) Å calculated for pristine Bi_2_Te_3_ from the data of reference [36]; the increase in covalency within the quintuple layers seems to imply a decrease of the interlayer interactions, with a significant separation of adjacent covalent blocks.

### Nanostructuration

**Fig. 3:**
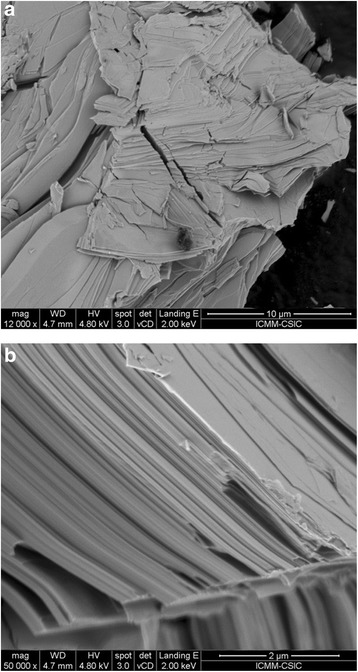
SEM images displaying the superficial morphology of n-Bi_2_Te_2.4_Se_0.6_, formed by stacked nanosized flakes (perpendicular to [001] direction). **a** ×12,000 and **b** ×50,000 magnification, where distinctive sheet thickness is around 25 nm

Figure 3a, b displays the superficial morphology of the as-grown Bi_2_Te_2.4_Se_0.6_ pellets collected with increasing magnification (×12,000 and ×50,000, respectively) in SEM. The sample is formed of stacked nanosized sheets, each of them apparently single crystalline, with the large surfaces perpendicular to the *c* crystallographic axis. The characteristic cleavage of this material is apparent, a consequence of weak bonding between quintuple layers. The typical thickness of individual sheets is around 25 nm. The low thermal conductivity of this material produced by arc melting is probably related to this nanostructuration into separate sheets providing many surface boundaries, which increases phonon scattering.

### Transport measurements

**Fig. 4:**
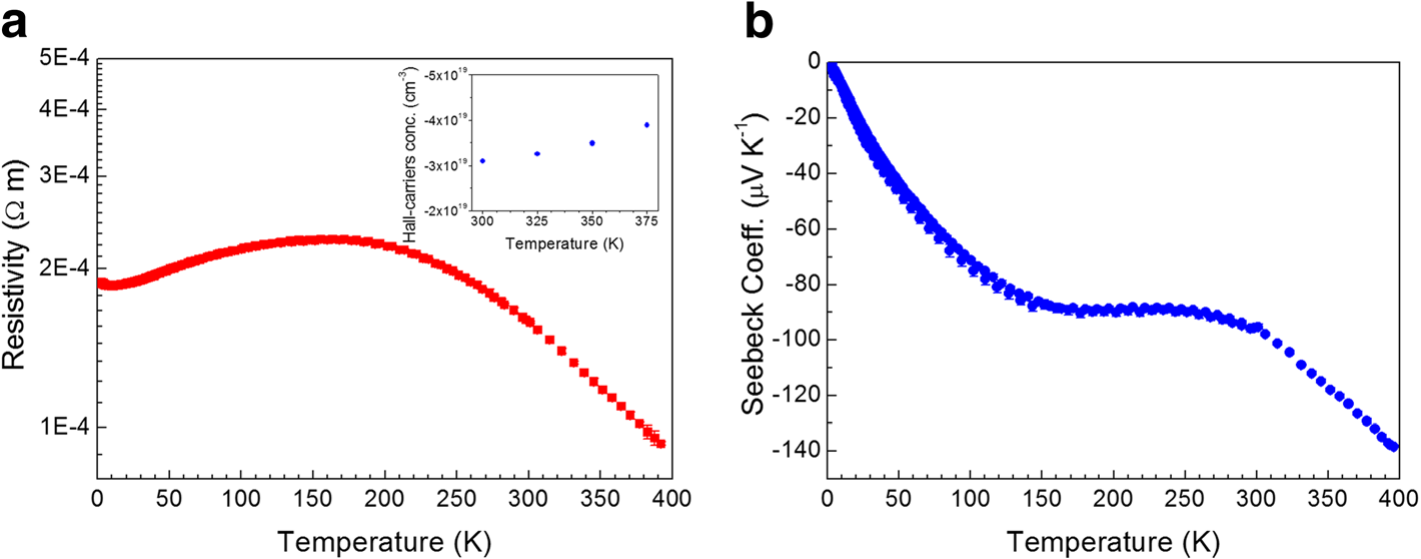
**a** Temperature dependence of the electrical resistivity of n-Bi_2_Te_2.4_Se_0.6_, showing the characteristic semimetallic behavior in the 2–400 K temperature range. The *inset* shows thermally excited carrier concentration determined by Hall effect. **b** Seebeck coefficient vs temperature

The temperature-dependent electrical resistivity is shown in Fig. 4a. The sample shows semimetallic behavior in the sense that its resistivity at low temperatures increases with temperature, as for a metal, but at higher temperatures it decreases, as for a semiconductor, until reaching a minimum measured value of 100 μΩ m at 365 K. For arc-melted Bi_2_Te_3_, resistivity values as low as 2 μΩ m are found at 320 K [36], while for samples prepared by other physical and wet chemical methods, resistivity values are around 30 and 5 μΩ m [40]. Therefore, in Se-doped Bi_2_Te_3_ arc melting leads to high electrical resistivity, even though for the undoped compound we obtained an improvement in the electrical conductivity [36]. These results are related to the scattering of carriers in grain boundaries and the point defects introduced by the random distribution of Te and Se in the crystalline positions. Soni et al. [39] found similar effects on carrier scattering, doping, and electrical conductivity for a Bi_2_Te_2.2_Se_0.8_ nanocomposite, with both metallic and semiconductor behavior throughout their measurement range with values around 75 μΩ at room temperature. By encapsulating melting and hot pressing, this value is reduced down to 12–20 μΩ m [41], while after SPS treatments, values close to 15 μΩ m were reported [35].

The n-type Hall-carrier concentration (inset of Fig. 4a) increases with increasing temperature due to thermal excitation, typical of semiconducting behavior. We find the charge carrier density at 300 K to be −3.1∙10^19^ cm^−3^, slightly higher than pure Bi_2_Te_3_ [36, 42]. The mobility is quite low at 300 K, determined using *μ*_H_ = *R*_H_ * *σ*, which results in 10.1 cm^2^ V^−1^ s^−1^ [35, 41]. Bi_2_Te_3_-type compounds are extremely anisotropic, where electron mobility is heavily influenced by the grain orientation [43].

The Seebeck coefficient vs temperature is shown in Fig. 4b. The n-type Seebeck coefficient progressively increases between 2 and 400 K, reaching −140 μV K^−1^ at 390 K. The plateau in the Seebeck coefficient is related to the maximum found in the electrical resistivity. We can think of two effects that may explain this behavior: Thermal excitation of carriers diminishes bipolar transport at higher temperatures, causing a shift of the temperature dependence of the Seebeck coefficient, too. Also, potential barrier scattering, a thermally activated process that increases the Seebeck coefficient, may play an important role in the highly granular material produced by arc melting [44]. These results were checked in numerous samples. In comparison, the parent compound Bi_2_Te_3_ shows similar n-type semimetallic behavior, with reported values in the range of −50 to −260 μV K^−1^ for samples prepared by different chemical and physical methods [15, 42, 45]. In particular, arc melting yields samples with around −50 μV/K [36]. For Se-doped samples, similar Seebeck coefficients at 300 K close to −150 μV K^−1^ are reported [41]; much better values are reported by Soni et al. [39] after tuning the Se composition, where the optimal thermoelectric performance has been found in Bi_2_Te_2.7_Se_0.3_ nanocomposite, which exhibits its best Seebeck coefficient value of −259 μV/K at room temperature.

**Fig. 5:**
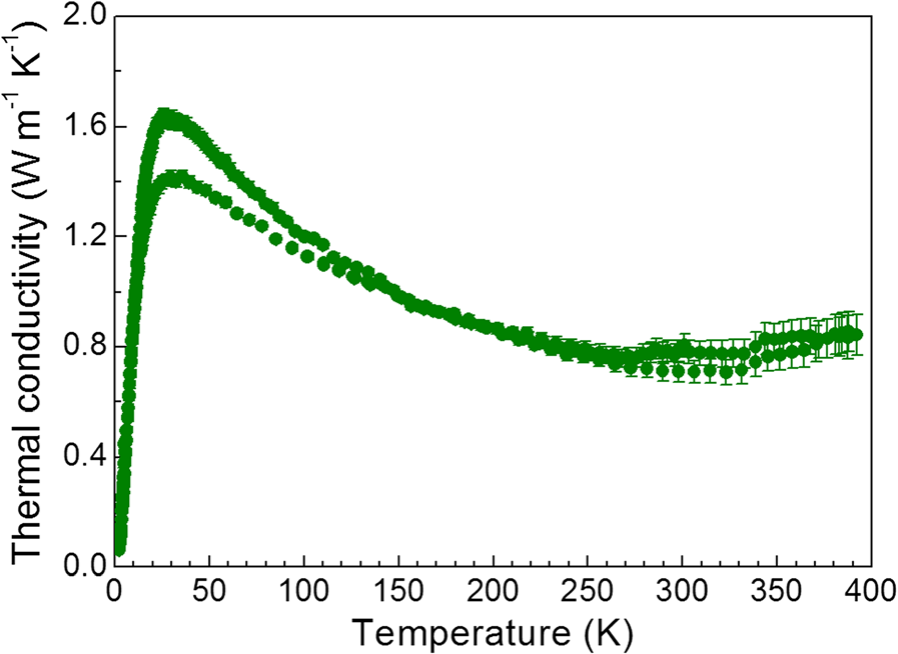
Thermal conductivity vs temperature of n-Bi_2_Te_2.4_Se_0.6_

Figure 5 displays the evolution of total thermal conductivity vs temperature. At low temperature, it shows the expected Umklapp maximum and then it decreases throughout the measurement range to a minimum value of 0.8 W m^−1^ K^−1^ at room temperature. This is one of the best (lowest) values for the Bi_2_Te_3_ system [15], typically above 0.9 W m^−1^ K^−1^. This value is lower than that obtained in our previous study for nanostructured Bi_2_Te_3_ obtained by arc melting, where thermal conductivity reached 1.2 W m^−1^ K^−1^ at 365 K [36]. This reduction could be related to the higher anisotropy and electrical resistivity, nanostructuration, and point defects induced by Se doping. The underlying reason is likely the strong phonon scattering at grain boundaries associated with the sheet-type nanostructuration. For the nanostructured parent compound Bi_2_Te_3_ prepared by ball milling and hot pressing, measured thermal conductivities were 1.2 W m^−1^ K^−1^ at 330 K [46], and for chemically prepared samples, the thermal conductivity values are around 0.8 W m^−1^ K^−1^ at 380 K [45].

Within the Bi_2_Te_3_-Bi_2_Se_3_ system, for solid solutions prepared by encapsulated melting and hot pressing, typical thermal conductivity values of 1.04 W m^−1^ K^−1^ at 323 K have been reported [41]; for samples prepared by large-scale zone melting *κ* = 1.2 W m^−1^ K^−1^ at 323 K [44]. In the case of nanocomposite materials prepared by the polyol method and SPS, much lower values of thermal conductivity are found such as 0.9 W m^−1^ K^−1^ at 300 K for Bi_2_Te_2.2_Se_0.8_ nanocomposites and exceptionally 0.7 W m^−1^ K^−1^ at 300 K for Bi_2_Te_2.7_Se_0.3_ nanocomposites [39], but these fabrication techniques are, by far, more complex and time-consuming than the arc melting presented here.

## Conclusions

A Se-doped Bi_2_Te_3_ specimen of composition Bi_2_Te_2.4_Se_0.6_ has been synthesized by a straightforward arc-melting technique, yielding highly nanostructured samples in short reaction times, with improved thermal transport properties. A structural NPD study yields interesting hints on the increased covalency of the quintuple layers and the weakening of the interactions between adjacent layers upon Se doping. As a consequence of this structural feature, the trend to cleave and to form nanostructured specimens increases with respect to the pristine Bi_2_Te_3_ alloy. The Se doping (located at both Te1 and Te2 crystallographic sites, as shown from NPD data) enhances the carrier scattering, thus diminishing the electrical conductivity and results in low mobility. The Bi_2_(Te_1 − *x*_Se_*x*_)_3_ system is notably affected by the nanostructuration which leads to improved (lower) thermal conductivities.

## Abbreviations

2D: Two dimensional
FE-SEM: Field effect scanning electron microscope
HRPT: High-resolution powder diffractometer for thermal neutrons
NPD: Neutron powder diffraction
PPMS: Physical property measurement system
PSI: Paul Scherrer Institute
R_H_: Hall coefficient
RT: Room temperature
*S*: Seebeck coefficient
SEM: Scanning electron microscopy
SINQ: The Swiss spallation neutron source
SPS: Spark plasma sintering
*T*: Temperature
XRD: X-ray diffraction
ZT: Thermoelectric figure of merit
*κ*: Thermal conductivity
μ_H_: Hall mobility
*σ*: Electric conductivity
